# Assessing the function of STAS domain protein SypA in *Vibrio fischeri* using a comparative analysis

**DOI:** 10.3389/fmicb.2015.00760

**Published:** 2015-07-28

**Authors:** Cecilia M. Thompson, Karen L. Visick

**Affiliations:** Department of Microbiology and Immunology, Loyola University Chicago, Maywood, ILUSA

**Keywords:** *Vibrio fischeri*, *Vibrio parahaemolyticus*, *Vibrio vulnificus*, biofilm formation, STAS domain

## Abstract

Colonization of the squid *Euprymna scolopes* by *Vibrio fischeri* requires biofilm formation dependent on the 18-gene symbiosis polysaccharide locus, *syp*. One key regulator, SypA, controls biofilm formation by an as-yet unknown mechanism; however, it is known that SypA itself is regulated by SypE. Biofilm-proficient strains form wrinkled colonies on solid media, while *sypA* mutants form biofilm-defective smooth colonies. To begin to understand the function of SypA, we used comparative analyses and mutagenesis approaches. *sypA* (and the *syp* locus) is conserved in other *Vibrios*, including two food-borne human pathogens, *Vibrio vulnificus* (*rbdA*) and *Vibrio parahaemolyticus* (*sypA_*VP*_*). We found that both homologs could complement the biofilm defect of the *V. fischeri sypA* mutant, but their phenotypes varied depending on the biofilm-inducing conditions used. Furthermore, while SypA_VP_ retained an ability to be regulated by SypE, RbdA was resistant to this control. To better understand SypA function, we examined the biofilm-promoting ability of a number of mutant SypA proteins with substitutions in conserved residues, and found many that were biofilm-defective. The most severe biofilm-defective phenotypes occurred when changes were made to a conserved stretch of amino acids within a predicted α-helix of SypA; we hypothesize that this region of SypA may interact with another protein to promote biofilm formation. Finally, we identified a residue required for negative control by SypE. Together, our data provide insights into the function of this key biofilm regulator and suggest that the SypA orthologs may play similar roles in their native *Vibrio* species.

## Introduction

Bacteria encounter a variety of environments, not all of which are favorable, and have adopted many strategies to survive unfavorable environmental conditions. One survival strategy bacteria employ is the formation of a biofilm, a complex community of microorganisms encased in an extracellular matrix comprised of polysaccharides, protein, and extracellular DNA (Sutherland, 2001; Fux et al., 2005). Biofilms have been observed in nature, in industrial settings, and in healthcare, where biofilms have been found on catheters and other indwelling medical devices. Bacteria protected by a biofilm exhibit increased resistance to antibiotics and antimicrobials (Donlan, 2001). Biofilm formation in the context of a human host can therefore have serious consequences in the clinical setting (Donlan, 2001).

One model system used to study host-relevant biofilm formation is *Vibrio fischeri* and its symbiotic host, the Hawaiian bobtail squid *Euprymna scolopes* (Stabb and Visick, 2013; McFall-Ngai, 2014). The ability of this marine bacterium to efficiently colonize *E. scolopes* depends on the formation of a biofilm (Nyholm et al., 2000; Yip et al., 2006; Morris et al., 2011). Biofilm formation by *V. fischeri* depends, in part, on expression of the 18-gene symbiosis polysaccharide locus (*syp*), which encodes biofilm regulators as well as the proteins necessary for the production and export of the polysaccharide component of the biofilm (Supplementary Figures S1 and S2; Yip et al., 2005, 2006). Mutations in genes of the *syp* locus abrogate biofilm formation and disrupt colonization (Yip et al., 2005; Shibata et al., 2012).

The process of forming the biofilm is regulated at multiple levels, including transcription and a poorly understood post-transcriptional control mechanism. Transcription of the *syp* locus is controlled by an unusual and complex two-component signaling cascade comprised of two hybrid sensor kinases, RscS and SypF, and a response regulator, SypG (Supplementary Figure S1A; Visick and Skoufos, 2001; Yip et al., 2006; Hussa et al., 2008; Norsworthy and Visick, 2015). Upon sensing an as-yet-unknown signal, RscS autophosphorylates and initiates a phosphorelay: the phosphoryl group is transferred sequentially to SypF and then to the DNA binding protein SypG, which activates transcription of the *syp* locus (Ray et al., 2013). The result is production of Syp proteins and, ultimately, of the Syp polysaccharide (Syp-PS; Yip et al., 2005, 2006).

The RscS-mediated phosphorelay also controls the phosphorylation state of SypE (Supplementary Figure S1A; Morris et al., 2011; Norsworthy and Visick, 2015). SypE is a complex response regulator with two output domains that have opposing functions. Depending on its activation state, SypE functions as either a serine kinase or a serine phosphatase (Morris et al., 2011; Morris and Visick, 2013a,b). Unphosphorylated SypE functions as a kinase to phosphorylate and inactivate its target, SypA, on a conserved serine residue, S56 (Morris and Visick, 2013a,b). When signaled via the RscS phosphorelay, SypE functions as a phosphatase to dephosphorylate and activate the same target.

In the laboratory, biofilm formation is readily observed on plates by the production of wrinkled colonies (as opposed to smooth colonies), and can be induced in two ways: overexpression of the sensor kinase, *rscS,* or overexpression of the response regulator, *sypG*. RscS promotes biofilm formation by activating both SypG to induce *syp* transcription and SypE to dephosphorylate SypA; thus, overexpression of *rscS* alone is sufficient for biofilm formation (Supplementary Figure S1A). In contrast, while overexpression of *sypG* promotes *syp* transcription, it does not promote SypE-dependent activation of SypA (Supplementary Figure S1B). Thus, to induce biofilm formation, the biofilm inhibitor *sypE* must be deleted in strains overexpressing *sypG* (Supplementary Figure S1C; Hussa et al., 2008).

Encoded by the first gene in the *syp* locus, SypA plays a critical role in regulating biofilm formation. Deletion of *sypA* prevents biofilm formation and squid colonization (Morris et al., 2011). These defects can be complemented by a wild-type copy of *sypA* or by *sypA*-S56A, which encodes a non-phosphorylatable form of SypA, but not by *sypA*-S56D, which encodes a phospho-mimic. Thus, SypA is in its active state when unphosphorylated. Although SypA is a key regulator of biofilm formation, the mechanism by which SypA functions remains to be determined.

SypA contains a single STAS (anti-sigma factor antagonist and sulfate transporter) domain (Morris and Visick, 2010; Sharma et al., 2011). Frequently found within multi-domain proteins involved in signal transduction and transport, such as sulfate transporters (Shibagaki and Grossman, 2006; Marles-Wright et al., 2008), STAS domains are also found in single-domain proteins encoded in numerous bacterial genomes. Notably, the single STAS domain proteins RsbV and SpoIIAA have been well-studied for their roles as anti-sigma factor antagonists in *Bacillus subtilis* (e.g., Dufour and Haldenwang, 1994; Yudkin and Clarkson, 2005). Like SypA, RsbV, and SpoIIAA contain a conserved serine residue that is phosphorylated and controls protein activity. Despite the overall similarities between these proteins, no evidence to date suggests that SypA also acts as an anti-sigma factor antagonist (Morris and Visick, 2010, 2013b). Indeed, there are many single domain STAS proteins whose functions in various cellular processes appear to be distinct from the well-studied *Bacillus* proteins, such as the BtrV STAS domain protein from *Bordetella*, which plays a role in type III secretion (Mattoo et al., 2004). The exact function of BtrV is also unknown.

Orthologs of SypA can be found encoded within a conserved *syp* locus in other *Vibrio* species, including the human pathogens *Vibrio vulnificus* (RbdA) and *Vibrio parahaemolyticus* (SypA_VP_; Supplementary Figure S2). In *V. vulnificus*, the orthologous locus, *rbd*, also plays a role in biofilm formation (Guo and Rowe-Magnus, 2011). Specifically, when expression of the locus is induced, *V. vulnificus* exhibits increased biofilm phenotypes, including a cell-clumping/auto-aggregation phenotype. The role of RbdA in this phenotype has not yet been reported. However, the pathway for post-transcriptional control may be different in *V. vulnificus* and *V. parahaemolyticus*, as neither organism contains an ortholog of *sypE*, making it unclear if either RbdA or SypA_VP_ is controlled via phosphorylation like SypA.

Here, we investigated the structure/function of SypA using two approaches. First, we asked if the function of SypA was conserved in the *V. vulnificus* and *V. parahaemolyticus* homologs by investigating their ability to complement a *V. fischeri sypA* mutant. Then, we mutated residues conserved among the three proteins and asked if any were required for function of the *V. fischeri* protein. Together, these studies give insight into the requirements for SypA to function in *V. fischeri* and suggest that the SypA-like proteins may function similarly in their own species.

## Materials and Methods

### Strains and Media

*Vibrio fischeri* strains used in this study are listed in **Table 1**, and plasmids used are listed in Supplementary Table S1. *V. fischeri* strains were derived by conjugation. *Escherichia coli* GT115 (Invivogen, San Diego, CA, USA), π3813 (Le Roux et al., 2007) and S17-1λ*pir* were used (Simon et al., 1983) for cloning and conjugation experiments (Boettcher and Ruby, 1990; Visick and Skoufos, 2001). *V. fischeri* strains were cultured in Luria-Bertani salt (LBS) medium (Stabb et al., 2001). The following antibiotics were added to LBS medium at the indicated concentrations: chloramphenicol (Cm) 2.5 μg ml-1, erythromycin at 5 μg ml-1, and tetracycline (Tet) at 5 μg ml-1. *E. coli* strains were cultured in Luria-Bertani medium (LB; Davis et al., 1980). The following antibiotics were added to LB medium at the indicated concentrations: kanamycin (Kan) at 50 μg ml-1, Tc at 15 μg ml-1, or ampicillin (Ap) at 100 μg ml-1. For solid media, agar was added to a final concentration of 1.5%.

**Table 1:** *Vibrio fischeri* strains used in this study.

Strains	Genotype	Source or reference
KV4715	Δ*sypA*	Morris and Visick (2013b)
KV4716	Δ*sypA* Δ*sypE*	Morris and Visick (2013b)
KV5079	Δ*sypA* attTn7::*erm^*R*^*	Morris and Visick (2013b)
KV5479	Δ*sypA* attTn*7*::*sypA*	Morris and Visick (2013b)
KV5481	Δ*sypA* attTn*7*::*sypA^*S56A*^*	Morris and Visick (2013b)
KV6392	Δ*sypA* Δ*sypE* attTn*7*::*erm^*R*^*	Morris and Visick (2013b)
KV6393	Δ*sypA* Δ*sypE* attTn*7*::*sypA*	Morris and Visick (2013b)
KV6578	Δ*sypA* attTn*7*::*sypA*-HA	Morris and Visick (2013a)
KV6579	Δ*sypA* attTn*7*::*sypA^*S56A*^* HA	Morris and Visick (2013a)
KV6580	Δ*sypA* Δ*sypE* attTn*7*::*sypA*-HA	Morris and Visick (2013a)
KV6995	Δ*sypA* attTn*7*::*sypA^*K67A*^* HA	This study
KV7000	Δ*sypA* attTn*7*::*sypA^*Q84A*^* HA	This study
KV7005	Δ*sypA* attTn*7*::*sypA^*R68A*^* HA	This study
KV7010	Δ*sypA* attTn*7*::*sypA^*R93A*^* HA	This study
KV7309	Δ*sypA* attTn*7*::*rbdA*	This study
KV7310	Δ*sypA* Δ*sypE* attTn*7*::*rbdA*	This study
KV7313	Δ*sypA* attTn*7*::*sypA_*VP*_*	This study
KV7314	Δ*sypA* Δ*sypE* attTn*7*::*sypA_*VP*_*	This study
KV7315	Δ*sypA* attTn*7*::*sypA_*VP*_-*HA	This study
KV7316	Δ*sypA* Δ*sypE* attTn*7*::*sypA_*VP*_-*HA	This study
KV7558	Δ*sypA* attTn*7*::*sypA^*E2A*^* HA	This study
KV7560	Δ*sypA* attTn*7*::*sypA^*G25A*^* HA	This study
KV7562	Δ*sypA* attTn*7*::*sypA^*D34A*^* HA	This study
KV7564	Δ*sypA* attTn*7*::*sypA^*Y64A*^* HA	This study
KV7566	Δ*sypA* attTn*7*::*sypA^*E71A*^* HA	This study
KV7568	Δ*sypA* attTn*7*::*sypA^*R74A*^* HA	This study
KV7570	Δ*sypA* attTn*7*::*sypA^*G83A*^* HA	This study
KV7572	Δ*sypA* attTn*7*::*sypA^*P99A*^* HA	This study
KV7606	Δ*sypA* attTn*7*::*sypA^*D47A*^* HA	This study
KV7607	Δ*sypA* attTn*7*::*sypA^*D55A*^* HA	This study
KV7612	Δ*sypA* attTn*7*::*sypA^*D73A*^* HA	This study
KV7613	Δ*sypA* attTn*7*::*sypA^*R27A*^* HA	This study
KV7615	Δ*sypA* attTn*7*::*sypA^*Y66A*^* HA	This study
KV7616	Δ*sypA* attTn*7*::*sypA^*K72A*^* HA	This study
KV7620	Δ*sypA* attTn*7*::*sypA^*K90A*^* HA	This study


### Bioinformatics

Amino acid sequences for *V. vulnificus* RbdA (VV1_2658, WP_011080500), *V. parahaemolyticus* SypA_VP_ (VP1476, NP_797855.1), and *V. fischeri* SypA (VF_A1020, YP_206978.1) were obtained from the National Center for Biotechnology Information (NCBI) database. Alignments of RbdA, SypA_VP_, and SypA were generated using BLAST and the Clustal Omega multiple-sequence alignment program from EMBL-EBI (http://www.ebi.ac.uk/Tools/msa/clustalw2/; Altschul et al., 1997, 2005; Larkin et al., 2007; Sievers et al., 2011).

### Molecular and Genetic Techniques

The *rbdA* and *sypA_VP_* alleles used in this study were generated, and, in some cases, HA epitope-tagged, by polymerase chain reaction (PCR) using primers listed in Supplementary Table S2 and DNA from *V. vulnificus* strain ATCC29307 and *V. parahaemolyticus* strain RIMD2210633 (KXV237), respectively. The PCR products were cloned, using the Gibson Assembly kit (New England Biolabs), into plasmid pARM47 that was digested to remove the *sypE* gene. pARM47 provides the *lac* promoter and facilitates chromosomal insertion at the Tn*7* insertion site. To generate site-directed mutations in *sypA*, mutated alleles of *sypA* were generated by PCR using mutagenic primers (Supplementary Table S2) and plasmid pARM163 as a template and cloned into pARM47 using Gibson assembly as described above. The mutations were confirmed by sequence analysis using ACGT, inc. (Wheeling, IL, USA). All pARM47-based constructs contain two promoters, the vector-containing *lac* promoter and the *sypA* promoter, and were inserted into the chromosomal Tn*7* site of *V. fischeri* strains using tetraparental conjugation (McCann et al., 2003).

### Wrinkled Colony Formation Assay

To observe wrinkled colony formation, the indicated *V. fischeri* strains were streaked onto LBS agar plates containing the necessary antibiotics. Single colonies were then cultured with shaking in LBS broth containing antibiotics overnight at 28°C. The strains were then sub-cultured the following day in 5 ml of fresh medium. Following growth to early log phase, the cultures were standardized to an optical density at 600 nm (OD_600_) of 0.2 using LBS. 10 μl of diluted cultures were spotted onto LBS agar plates containing necessary antibiotics, and grown at either ∼24°C (for *rscS* overexpressing strains) or 28°C (for *sypG* overexpressing strains). At the end of the time course, the colonies were disrupted with a toothpick to assess colony cohesiveness, which is an indicator of Syp-PS production (Ray et al., 2015). Images of the spotted cultures were acquired over the course of wrinkled colony formation at the indicated times using a Zeiss Stemi 2000-C dissecting microscope.

### Western Blot Analysis of *V. fischeri* Lysates

*Vibrio fischeri* strains were cultured in LBS containing the appropriate antibiotics overnight at 25°C. Cultures (1 ml) were standardized to an OD_600_ equal to 3, concentrated by centrifugation, and lysed in 200 μl 2X sample buffer (4% SDS, 40 mM Tris pH 6.3, 10% glycerol). Samples were resolved on 12% TruPAGE^TM^ Precast gels (Sigma-Aldrich, St. Louis, MO, USA), and transferred to PVDF membranes. SypA and SypA_VP_ were detected by Western blot analysis using rabbit anti-HA antibody (Sigma-Aldrich, St. Louis, MO, USA) followed by a secondary, donkey anti-rabbit IgG antibody (Sigma-Aldrich, St. Louis, MO, USA) conjugated to horseradish peroxidase (HRP), and visualized using SuperSignal West Pico Chemiluminescent Substrate (Thermo Fischer Scientific, Rockford, IL, USA).

## Results

### The Conservation of SypA Among Select *Vibrio* Species

**FIGURE 1:**
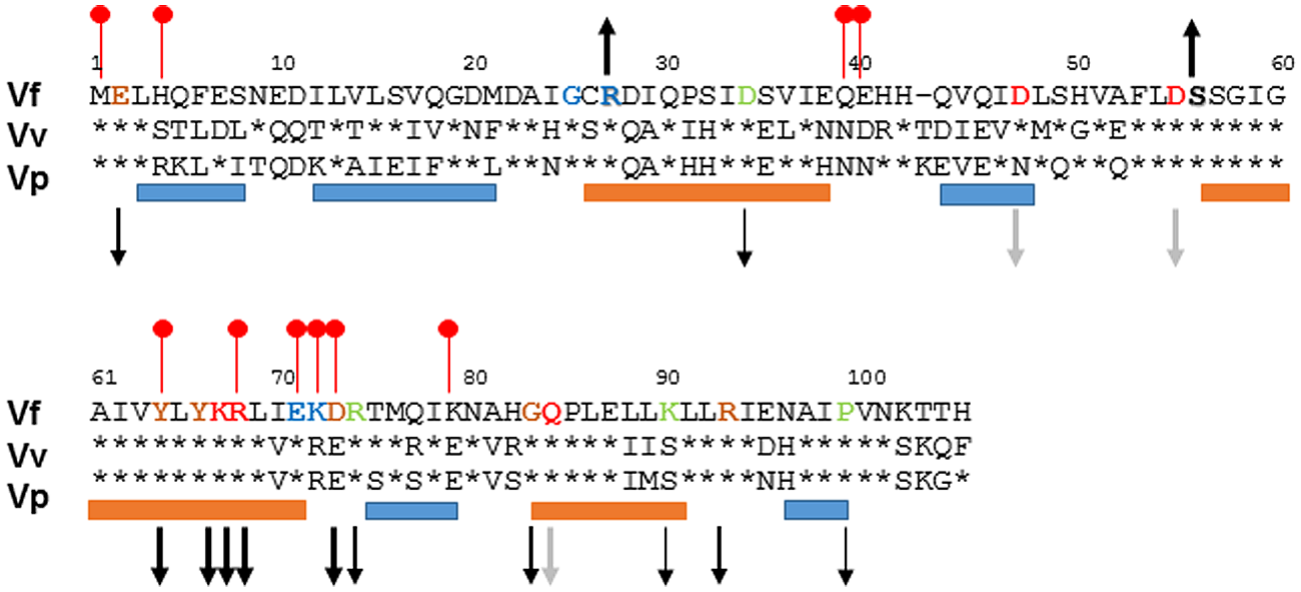
**Alignment of SypA orthologs.** To determine the similarity between SypA, RbdA, and SypA_VP_, we aligned the three proteins using ClustalW2 (Larkin et al., 2007). Residues that are identical to *Vibrio fischeri* SypA are shown as asterisks. The amino acid sequence is highly conserved around serine 56, depicted in bold lettering, which in *V. fischeri* SypA is phosphorylated by SypE. Up and down arrows indicate the biofilm phenotype (increased or decreased) that occurred when the indicated residues were changed to alanine, with the thickness of the arrows corresponding to the severity of the effect (thicker arrows, more severe); black arrows, SypA protein detectable; gray arrows, SypA protein undetectable or severely reduced. The orange and blue bars represent with helix and strand regions, respectively, predicted by PredictProtein (Rost et al., 1996, 2004), while the red lollypops represent putative protein interaction sites (Ofran and Rost, 2007). Residues that have been mutated are colored: blue for Class I mutants, green for Class II mutants, orange for Class III, and red for Class IV mutants.

To better understand the function of SypA, we used BLAST and ClustalW2 analyses (Altschul et al., 1997, 2005; Larkin et al., 2007) to compare SypA to the SypA orthologs encoded within loci similar to the *syp* locus in *V. vulnificus* (RbdA) and *V. parahaemolyticus* (SypA_VP_). Relative to SypA, RbdA exhibited 55% identity and 73% similarity, while SypA_VP_ exhibited 58% identity and 73% similarity at the amino acid level (**Figure 1**). Both proteins contained the conserved serine that, in SypA, is phosphorylated (S56 in *V. fischeri*, S57 in *V. vulnificus* and *V. parahaemolyticus*). The three proteins were most similar in their central regions (near the conserved serine) and C-termini, and most dissimilar in their N-termini. We hypothesize that, due to the high sequence similarity between the SypA proteins, their function in promoting biofilm formation might also be conserved.

### The Function of *V. fischeri* SypA is Conserved Among *Vibrio* Species

**FIGURE 2:**
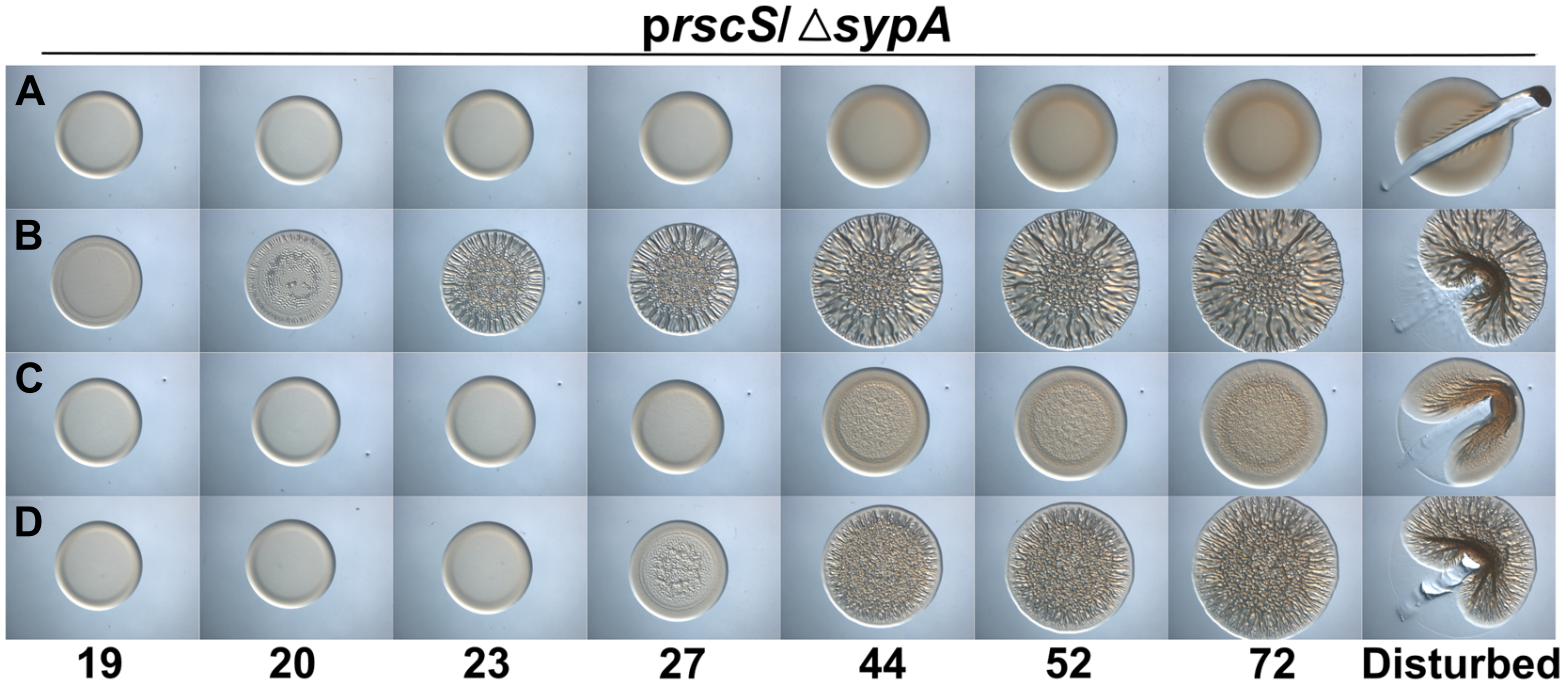
***rbdA* and *syp*A*_*VP*_* promote Syp-PS production when RscS is overexpressed.** Development of colony morphology over time of *rscS* (pARM7)-overexpressing derivatives of Δ*sypA* strains that contain **(A)** the empty cassette (negative control; KV5079), **(B)** *sypA* (KV5479), **(C)** *rbdA* (KV7309), or **(D)** *sypA_*VP*_* (KV7313). Cultures were spotted onto LBS plates containing tet, and the morphologies of the resulting colonies were assessed at the indicated times. Representative images are shown. At 72 h, the colonies were disturbed with a toothpick to assess colony cohesiveness.

To determine if the function of SypA is conserved despite the observed differences, we assessed the ability of the two non-native genes to complement a *V. fischeri sypA* mutant. First, we cloned the genes under the control of the native *V. fischeri sypA* promoter into a vector that also contained a *lac* promoter oriented such that it could drive transcription. Then, we introduced these constructs into the chromosome of a *sypA* deletion mutant and induced biofilm formation by overexpressing the sensor kinase *rscS* from a multi-copy plasmid. Finally, we evaluated biofilm formation by assessing the formation of wrinkled colonies using a spotted culture technique (see Materials and Methods). The negative control, an uncomplemented parent strain, never formed wrinkled colonies, while the positive control, a parent strain complemented with the native *V. fischeri sypA* gene, formed wrinkled colonies within 20 h (**Figures 2A,B**). Complementation with the *sypA* homologs restored biofilm formation to the *sypA* mutant, although not to the same extent as the positive control. The *V. fischeri* mutant that contained *rbdA* formed colonies with minimal 3D architecture, but only after a substantial delay (∼1 day) compared to the positive control (**Figure 2C**). The *sypA*_VP_-containing strain was more proficient at biofilm formation than the *rbdA* strain, forming more developed wrinkled colonies after a shorter, ∼7 h, delay (**Figure 2D**). However, even after prolonged incubation, the wrinkling pattern of the *sypA*_VP_-containing strain was less developed than that of the positive control.

To further assess the function of SypA_VP_ and RbdA, we evaluated the production of Syp-PS using a toothpick assay: the production of Syp-PS leads to colonies in which the cells become cohesive, even if the colonies don’t wrinkle (Ray et al., 2015). When we disrupted the colonies formed by *sypA*_VP_ and *rbdA*-expressing *V. fischeri*, we found that both strains formed colonies with cohesive properties similar to the positive control (**Figure 2**, right-hand images). Together, these data suggest that both RbdA and SypA_VP_ are SypA orthologs capable of promoting Syp-PS production and wrinkled colony formation.

### Different Biofilm Induction Conditions Impact Complementation by SypA Orthologs

**FIGURE 3:**
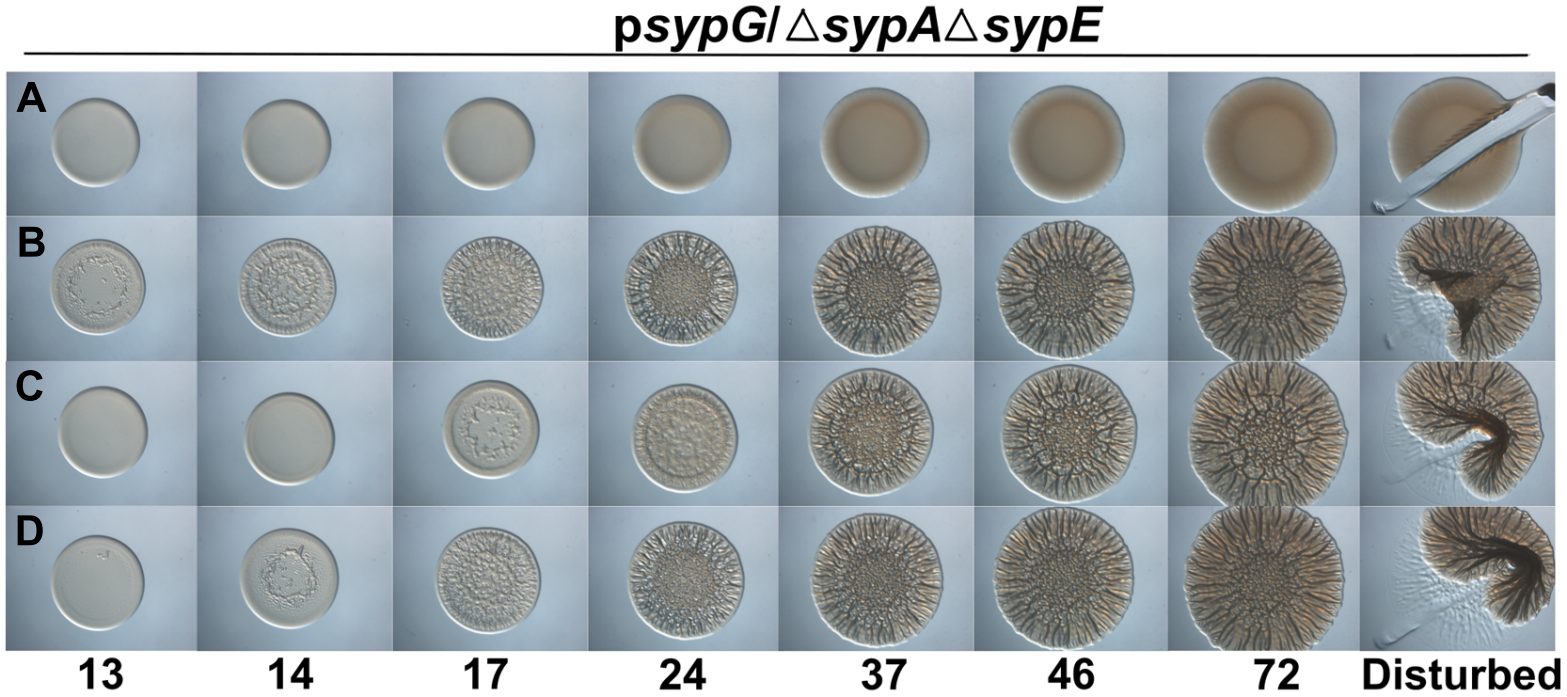
***rbdA* and *sypA_*VP*_* complement a *sypA* mutant for biofilm formation when *sypG* is overexpressed.** Development of colony morphology over time of *sypG* (pCLD56)-overexpressing derivatives of Δ*sypA* Δ*sypE* strains that contain **(A)** the empty cassette (negative control; KV6392), **(B)** *sypA* (KV6393), **(C)** *rbdA* (KV7310), or **(D)** *sypA*_VP_ (KV7314). Cultures were spotted onto LBS plates containing tet, and the morphologies of the resulting colonies were assessed at the indicated times. Representative images are shown. At 72 h, the colonies were disturbed with a toothpick to assess colony cohesiveness.

Because the activities of RbdA and SypA_VP_ were diminished relative to that of the native SypA protein under the conditions we used, we wondered if other methods of biofilm induction would permit better complementation. Specifically, we asked if overexpression of *sypG*, which encodes the response regulator that is the direct activator of the *syp* locus (Ray et al., 2013), would result in more robust biofilm formation. When *sypG* is overexpressed, the *syp* locus is highly induced, but biofilms do not form due to the activity of SypE (Supplementary Figures S1B,C). Therefore, we evaluated the activity of RbdA and SypA_VP_ in a *sypG*-overexpressing strain deleted for both *sypA* and *sypE*. As expected, the uncomplemented parent failed to form wrinkled colonies, while colonies of the *sypA*-complemented strain began wrinkling within 13 h (**Figures 3A,B**). In contrast to the poor complementation we observed above, under *sypG*-overexpressing conditions, both the *rbdA* and *sypA*_VP_-expressing strains formed wrinkled colonies with substantial 3D architecture after only a short (∼1–4 h) delay relative to the positive control (**Figures 3C,D**). At later times, the wrinkled colonies appeared indistinguishable from the positive control. Together, these data further suggest that the function of the SypA orthologs is conserved, but indicate that specific biofilm-induction conditions impact the resulting phenotype.

### Differences in Complementation are not due to Differences in Steady-State Protein Levels

**FIGURE 4:**
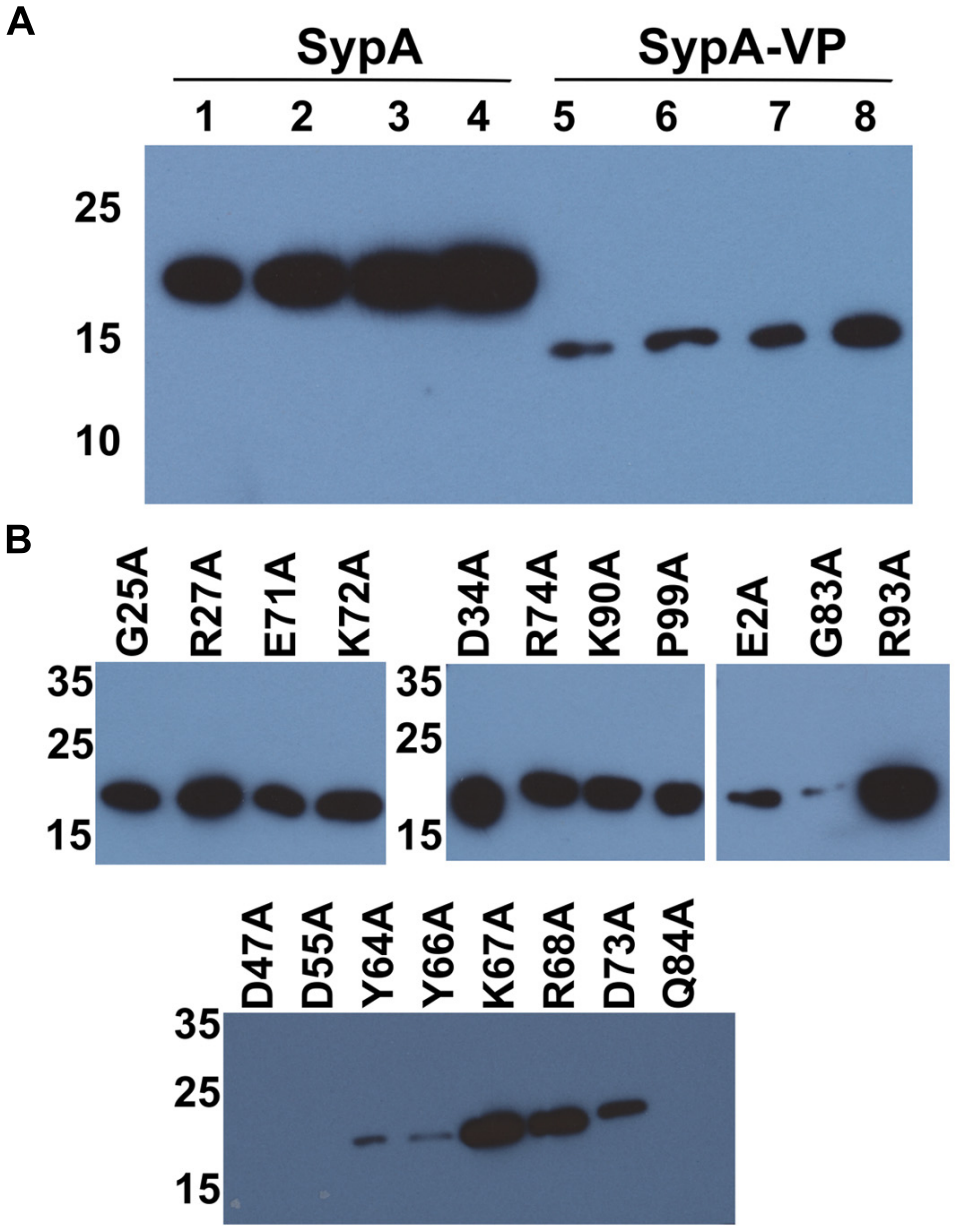
**Protein production under SypG- and RscS-induced biofilm conditions.** **(A)** HA-tagged derivatives of SypA (lanes 1–4) and SypA_VP_ (lanes 5–8) produced under *rscS*- (pARM7) or *sypG*- (pCLD56) overexpressing conditions and detected by Western blotting with anti-HA antibody. Lanes: 1 and 5, pARM7/Δ*sypA* (KV6578 and KV7315), 2 and 6, pARM7/Δ*sypA* Δ*sypE* (KV6580 and KV7316), 3 and 7, pCLD56/Δ*sypA* (KV6578 and KV7315) and 4 and 8, pCLD56/Δ*sypA* Δ*sypE* (KV6580 and KV7316). Lanes were loaded with 5 μl of whole cell lysate from cultures standardized to an OD_600_ = 3. **(B)** HA-tagged derivatives of mutant SypA proteins produced under *rscS*- (pARM7) overexpression conditions: G25A (KV7560); R27A (KV7613); E71A (KV7566); K72A (KV7616); D34A (KV7562); R74A (KV7568); K90A (KV7620); P99A (KV7572); E2A (KV7558); G83A (KV7570); R93A (KV7010); D47A (KV7606); D55A (KV7607); Y64A (KV7564); Y66A (KV7615); K67A (KV6995); R68A (KV7005); D73A (KV7612); Q84A (KV7000). All lanes were loaded with 5 μl of whole cell lysate from cultures standardized to an OD_600_ = 3.

The reason for the observed differences in complementation under the two biofilm-inducing conditions remains unclear. The two assays varied in several parameters, including temperature, the presence/absence of SypE, and the specific regulator used. We found that the differences were independent of temperature (data not shown). Also, the presence of SypE was not responsible for the poorer complementation, as complementation by *rbdA* was even worse in the *rscS* overexpression conditions when *sypE* was absent (Supplementary Figure S3); it is not clear why that is the case, although biofilm formation is slightly delayed even in otherwise wild-type *V. fischeri* strains deleted for *sypE* (Morris et al., 2011). Finally, we hypothesized that one difference between *rscS* and *sypG* overexpression conditions could be the strength of induction of SypG-dependent promoters, such that, potentially, more SypA is produced under *sypG* conditions. However, Western blot analysis of HA-epitope tagged versions of SypA and SypA_VP_ did not reveal dramatic differences between the two overexpression conditions (**Figure 4A**); thus, this explanation is unlikely to account for the observed differences in biofilm induction. We did note two differences between the SypA and SypA_VP_ samples: (1) The steady-state levels of SypA were substantially higher than those of SypA_VP_, regardless of the presence of SypE. Potentially, this difference could account for some, but not all, of the difference in complementation between *sypA* and its orthologs. (2) The two proteins migrated differently, with SypA_VP_ migrating as predicted from its molecular weight. The cause of this difference is unknown. It is formally possible that SypA forms a covalently bonded complex with itself or another protein, or else is modified by another molecule; if so, then such modifications are not critical for function, as SypA_VP_ was able to promote wrinkled colony formation.

### SypA_VP_ is Susceptible to Control by *V. fischeri* SypE

**FIGURE 5:**
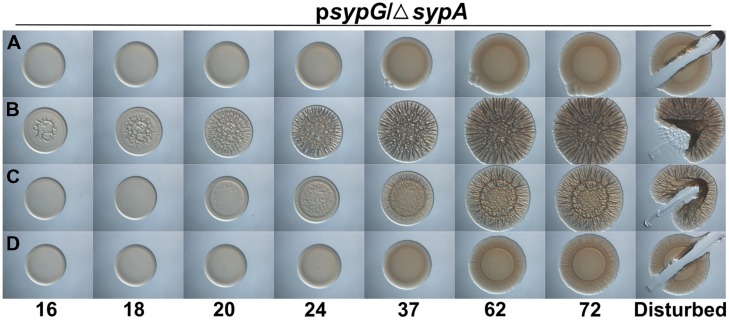
**RbdA and SypA_VP_ are subject to control by SypE.** Development of colony morphology over time of *sypG* (pCLD56)-overexpressing derivatives of Δ*sypA* (*sypE*+) strains that contain **(A)** native *sypA* (KV5479), **(B)** *sypA*-S56A (expressing a mutant SypA that cannot be phosphorylated by SypE; KV5481), **(C)** *rbdA* (KV7309), or **(D)** *sypA*_VP_ (KV7313). Cultures were spotted onto LBS plates containing tet, and the morphologies of the resulting colonies were assessed at the indicated times. Representative images are shown. At 72 h, the colonies were disturbed with a toothpick to assess colony cohesiveness.

The three proteins each contain a serine residue within a stretch of highly conserved residues; in SypA, this serine (S56) is phosphorylated by SypE (**Figure 1**; Morris and Visick, 2013a,b). Although *sypE* is missing from the chromosome of *V. vulnificus* and *V. parahaemolyticus*, it is possible that RbdA and SypA_VP_ are also controlled via phosphorylation and may retain the ability to interact with and be inactivated by SypE from *V. fischeri*. To determine if RbdA and SypA_VP_ were susceptible to inactivation by SypE, we expressed the *sypA* orthologs in a *sypE*-containing *sypA* mutant (Δ*sypA sypE*^+^) and induced biofilm formation by overexpressing *sypG*. As expected, the negative control, a parent strain complemented with wild-type SypA fully susceptible to phosphorylation, failed to form wrinkled colonies, while the positive control, a parent strain complemented with SypA^S56A^, a mutant that cannot be inactivated via phosphorylation, formed robust wrinkled colonies in less than 24 h (**Figures 5A,B**). Although initially smooth, the colonies formed by the *rbdA*-containing strain began to exhibit 3D architecture within 24 h, and were nearly as developed as the positive control at later times. When we disrupted the colonies formed by *rbdA*-expressing *V. fischeri*, we found that the strain formed colonies with cohesive properties, similar to the strain producing a mutant of SypA unable to be phosphorylated by SypE (**Figure 5C**). In contrast, the *sypA*_VP_-expressing strain failed to form wrinkled or cohesive colonies even at later times (**Figure 5D**). These data indicate that SypA_VP_ is susceptible to regulatory control by *V. fischeri* SypE, but that RbdA can largely overcome the inhibitory effect of SypE.

### Conserved Residues are Required for SypA Function

**FIGURE 6:**
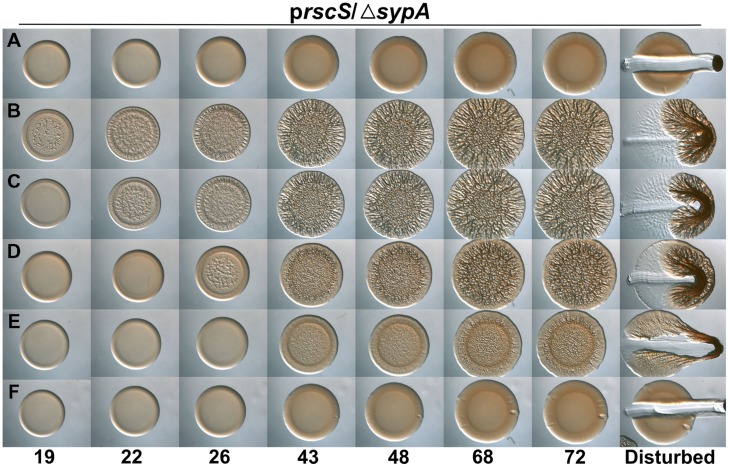
**Conserved residues in SypA function in biofilm formation.** Development of colony morphology over time of *rscS* (pARM7)-overexpressing derivatives of Δ*sypA* strains that contain **(A)** the empty cassette (negative control; KV5079), or expressing **(B)** *sypA* (KV6578), **(C)** *sypA*-R27A (representative Class I mutant; KV7613), **(D)** *sypA*-D34A (representative Class II mutant; KV7562), **(E)** *sypA*-R93A (representative Class III mutant; KV7010), or **(F)** *sypA*-K67A (representative Class IV mutant; KV6995). Cultures were spotted onto LBS plates containing tet, and the morphologies of the resulting colonies were assessed at the indicated times. Representative images are shown.

It remains unknown how SypA functions to promote biofilm formation, but we propose that unphosphorylated SypA interacts with a partner protein (other than SypE) to positively affect biofilm formation. Because *rbdA* and *sypA_VP_* were both able to promote biofilm formation in the absence of the native *sypA* gene, we hypothesized that amino acids important for interaction between SypA and its downstream partner are conserved in all three proteins (**Figure 1**). We thus chose 15 fully conserved residues, as well as four others that were conserved in two of the three organisms, and generated point mutations by substituting an alanine codon in place of the native *V. fischeri* codon. Then, we asked if these mutant *sypA* alleles could complement the biofilm defect of a *sypA* mutant. Similar results were observed in both *rscS*- and *sypG*-overexpressing conditions, and thus we show here the results obtained with the *rscS*-overexpressing conditions. Western blot analysis verified that 16 of the 19 mutant proteins were produced: the exceptions included SypA-Q84A, SypA-D47A, and SypA-D55A, which were undetectable; for three others, the amount of protein was severely reduced (SypA-Y64A, SypA-Y66A, and SypA-G83A; **Figure 4B**). Relative to the controls (**Figures 6A,B**), the mutant *sypA* alleles induced a range of wrinkled colony phenotypes. We thus divided the mutants into four classes based on the resulting phenotypes: I, not defective (4 of 19); II, delayed development (4 of 19); III, delayed and defective development (6 of 19); and IV, defective (little to no biofilm formation; 5 of 19). Representatives of these groups are shown in **Figure 6**, while the individual results are shown in Supplementary Figure S4.

Class I contained four strains that exhibited phenotypes indistinguishable, or nearly so, from the positive control. This class included strains expressing SypA proteins with the following substitutions: G25A, R27A, E71A, and K72A (**Figure 6C** and Supplementary Figure S4). Although there was a slight (∼1 h) difference in the start of wrinkled colony development for one of these mutants, the start of wrinkled colony development for the parent strain can vary up to 2 h. Therefore we conclude that the amino acid at positions 25, 27, 71, and 72 may not be critical for biofilm formation.

Class II contained four strains that exhibited a delay in colony development: SypA with alanine substitutions at D34, R74, K90, and P99. These strains formed wrinkled colonies with a morphology similar, but diminished, relative to the positive control within about 2 days (**Figure 6D** and Supplementary Figure S4). Of these, the strain that expressed D34A was the most developed. We conclude that these residues contribute to the efficiency of biofilm development under these conditions.

Class III included six of the 19 strains. These mutants were delayed and defective for biofilm formation: even after days of growth, these mutants never formed wrinkled colonies with 3D architecture similar to the positive control (**Figure 6E** and Supplementary Figure S4). The strains with this intermediate phenotype included those containing SypA with the following substitutions: E2A, Y64A, Y66A, D73A, G83A, and R93A. Although five of these mutants exhibited a reduction in the steady-state protein levels, which could account for their biofilm defects, the amount of SypA-R93A was similar to the steady-state levels of non-defective mutant SypA proteins. We conclude that R93A makes an important contribution to the formation of a mature biofilm.

The remaining five mutants were grouped in Class IV. When these mutant *sypA* alleles were introduced into *V. fischeri*, they resulted in strains completely abrogated for biofilm formation: the strains formed smooth colonies even after prolonged incubation (**Figure 6F** and Supplementary Figure S4). For three of these (D47A, D55A, and Q84A), we were unable to detect SypA protein by Western blot; likely, these mutations destabilize the SypA protein. However, the remaining strains produced stable proteins, a result that suggests that residues K67A and R68A are critical for the ability of SypA to induce biofilm formation. These mutant phenotypes are summarized in **Figure 1**.

### SypA Mutant R27A is Resistant to Control by SypE

**FIGURE 7:**
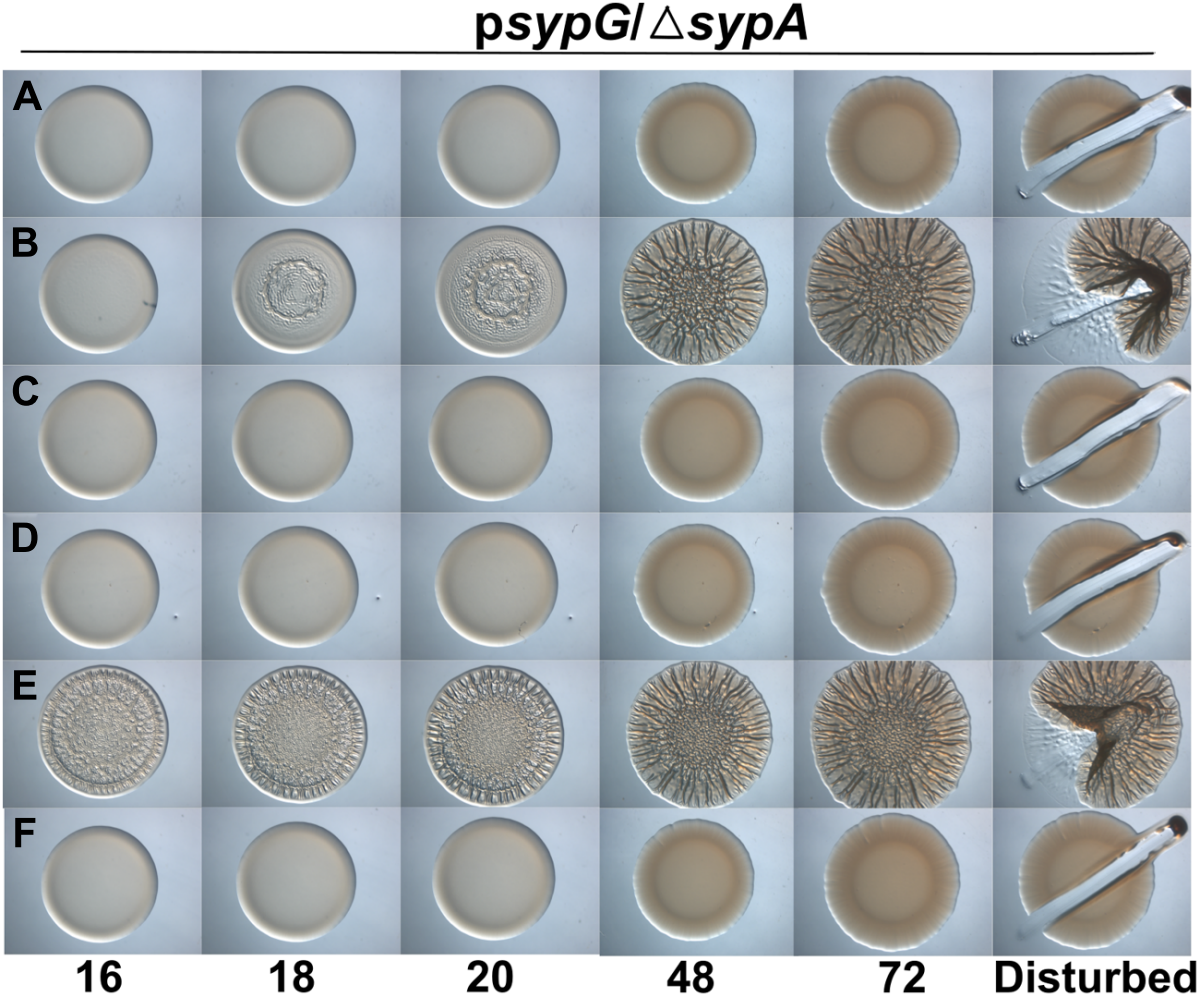
**SypA-R27A is resistant to control by SypE.** Development of colony morphology over time of *sypG* (pCLD56)-overexpressing derivatives of Δ*sypA* (*sypE*+) strains that contain **(A)** native *sypA* (susceptible to SypE; KV6587), **(B)** *sypA*-S56A (expressing a mutant SypA that cannot be phosphorylated by SypE; KV6579), or the Class I mutants as follows: **(C)** *sypA*- G25A (KV7560), **(D)** *sypA*-E71A (KV7566), **(E)** *sypA*-R27A (KV7613), and **(F)** *sypA*-K72A (KV7616). Cultures were spotted onto LBS plates containing tet, and the morphologies of the resulting colonies were assessed at the indicated times. Representative images are shown.

SypE binds to SypA and controls its activity via phosphorylation (Morris and Visick, 2013b). To date, Serine 56, the site of phosphorylation, is the only residue known to be critical for control by SypE. We hypothesized that other residues might facilitate the interaction between SypA and SypE, allowing for the phosphorylation of SypA, and that mutations in residues that facilitate this interaction would result in a SypA protein no longer recognized and/or phosphorylated by SypE. When introduced into a strain that expresses SypE, a mutant SypA that fails to interact with SypE will be “blind” to inhibition by SypE, resulting in biofilm formation under conditions in which it typically does not occur (e.g., *sypG* overexpression, Supplementary Figure S1B). We thus expressed the four Class I *sypA* mutant alleles (those able to promote wrinkled colony formation similar to the positive control **Figure 6C**) in a strain that contained *sypE* and induced biofilm formation by overexpressing *sypG*. As expected, the negative and positive control strains failed to form and formed, respectively, wrinkled colonies (**Figures 7A,B**). Not unexpectedly, three of the four mutants failed to induce wrinkled colony formation, indicating that the mutant SypA protein remained susceptible to inhibition by SypE. However, the fourth mutant, SypA-R27A, was able to promote biofilm formation even in the presence of SypE (**Figure 7E**). Interestingly, SypA-R27A induced wrinkled colony formation at least 3 h sooner than SypA-S56A, which cannot be phosphorylated by SypE. We conclude that R27 within SypA is important for the ability of SypE to inhibit SypA.

## Discussion

This study probed the structure/function relationship of *V. fischeri* SypA using comparative analyses and mutagenesis approaches. Orthologs of SypA (RbdA, SypA_VP_) encoded by other *Vibrio* species (*V. vulnificus*, *V. parahaemolyticus*) were able to complement the biofilm defect of a *V. fischeri sypA* mutant; one of these (SypA_VP_) also remained fully susceptible to inhibition by the regulatory protein SypE. These results indicated that the function of these proteins is conserved. Then, using the sequence identity as a guide, we mutated and assessed the requirement in biofilm formation of specific conserved residues, and found roles for a number of residues. In addition, we identified a specific residue required for control of SypA by SypE. These studies thus provide insight into the requirements for SypA function and control by SypE.

SypA is of interest because it plays a critical, yet unknown role in biofilm formation (Morris and Visick, 2013b). STAS domain proteins are abundant and play important roles in both prokaryotes and eukaryotes, yet remain poorly understood. Pfam currently shows that over 1900 STAS sequences have been identified in 364 eukaryotic species and over 8000 in more than 300 bacterial species. In bacteria, single-domain STAS proteins function as regulators of development and virulence (Kozak et al., 2005; Yudkin and Clarkson, 2005; Hecker et al., 2007). Although the best-characterized STAS domain proteins (e.g., *Bacillus* proteins SpoIIAA and RsbV Schmidt et al., 1990; Igoshin et al., 2006) function as anti-anti-sigma factors, other STAS domain proteins do not appear to function as anti-anti-sigmas (e.g., in *Chlamydia trachomatis* Hua et al., 2006 and *E. coli* YrbB Malinverni and Silhavy, 2009), but in distinct ways to control important cellular outputs such as type III secretion (Mattoo et al., 2004; Kozak et al., 2005). All of our evidence to date indicates that SypA also has a role other than as an anti-anti-sigma (Morris and Visick, 2013a,b). Thus, understanding the structure/function relationship of SypA has the potential not only to yield insight into the function of SypA but also potentially shed light on the roles of other STAS domain proteins in bacteria.

We initially chose to investigate SypA using a comparative analysis, with the dual goals of understanding SypA and, potentially, gaining insight into the function of the *sypA* orthologs in two pathogens, *V. parahaemolyticus* and *V. vulnificus*. We used *V. fischeri* because numerous tools exist and the wrinkled colony phenotype provides an easy visual for assessing function. Furthermore, to date, relatively little work has been done to investigate the *syp* (*rbd*) locus in these pathogens, although those studies support a role for it in polysaccharide production and biofilm formation. In *V. parahaemolyticus*, a recent study demonstrated a role for a *syp* gene (*sypQ*, which encodes a putative glycosyltransferase), in biofilm formation (attachment to glass) and host adherence functions, as well as in the production of poly-*N*-acetylglucosamine (PNAG; Ye et al., 2014). Although no other genes in the *V. parahaemolyticus syp* locus were investigated for their function in PNAG production or biofilm formation, the conserved function of *sypA*_VP_ in our studies suggests that it could contribute to PNAG production in *V. parahaemolyticus*. In *V. vulnificus*, the *rbd* locus contributes to biofilm formation, and in particular, cell–cell aggregation (Guo and Rowe-Magnus, 2011). It does not appear to contribute to the rugose (wrinkled) colony phenotype displayed by this organism under certain conditions; instead, this phenotype depends on a second polysaccharide locus, *brp* (Guo and Rowe-Magnus, 2011). The presence of two polysaccharide loci that contribute to different biofilm phenotypes suggests that *V. vulnificus* forms a different biofilm depending on the environment the bacterium encounters. Our results, that *rbdA* has a conserved function in promoting wrinkled colony formation, indicates that RbdA could perform a similar function to impact rugose colony formation in *V. vulnificus*; potentially, the role of *rbd* in promoting rugosity awaits identification of the right environmental (or genetic) conditions.

Our work revealed that both SypA_VP_ and RbdA could complement the *sypA* mutant. However, differences existed. In particular, SypA_VP_ induced the development of a more robust wrinkled colony than did RbdA, particularly under conditions in which *rscS* was overexpressed; this difference was even more apparent in the absence of SypE. Whether these differences can be attributed to the level of expression, stability, or activity of the SypA ortholog remains to be determined. Both orthologs exhibited a greater ability to complement when *sypG* was overexpressed. It is unclear why this is the case, as the apparent steady-state levels of SypA_VP_ under the two conditions was not dramatically altered. Understanding the basis for these differences may yield important insights into the function of SypA and the consequences of different modes of biofilm induction.

Using the similarities between the orthologs as a guide, we generated mutations in a number of conserved residues in SypA. Of the 19 alanine substitutions that we generated, two resulted in a non-functional but stably expressed protein. The two non-functional mutations, K67 and R68, as well as three delayed and defective mutations, Y64, Y66, and D73, were located in and near a predicted α-helix in the central, highly conserved portion of the protein (**Figure 1**). Given the high conservation, it is perhaps not surprising that this region is critical for function. We hypothesize that this helix is important for interacting with another protein to promote Syp-PS production and biofilm formation. In support of this possibility, PredictProtein (Rost et al., 2004), a program for protein structure predictions, lists five residues in this region, including Y64, R68 and D73, as putative protein binding residues (**Figure 1**; Ofran and Rost, 2007).

Biofilm formation was not impacted by alanine substitutions at positions G25, R27, E71, or K72 (Group I mutants). Two of these residues were fully conserved in RbdA and SypA_VP_, but positioned in the less-well conserved N-terminus. We hypothesized that the N-terminus could be important for interactions of SypA with SypE, and that mutations that prevent interaction of the two proteins could render SypA independent of control by SypE. Indeed, when we evaluated the ability of the four Group I mutants to promote biofilm formation in the presence of SypE, we found one mutant, SypA-R27A, that was able to do so. Of note, the SypE-expressing strain that contained SypA-R27A formed colonies that wrinkled sooner than the control strain that contained SypA-S56A, which is unable to be phosphorylated and thus inhibited by SypE (**Figure 7**). We hypothesize that SypE is unable to recognize and/or bind SypA-R27A; in contrast, while unable to phosphorylate SypA-S56A, SypE likely retains the ability to bind to this mutant, thus diminishing/delaying its activity. Analysis of R27 using PredictProtein (Rost et al., 2004) revealed that R27 is located on an exposed region of the first α-helix, a position that may be important for facilitating the interaction between SypA and SypE needed for the phosphorylation event.

Our studies suggested that SypE controls the activity of SypA_VP_ but not RbdA. Whether this control occurs via phosphorylation, as with SypA, or via another mechanism such as protein sequestration remains to be determined. SypA_VP_ and RbdA are more similar to each other than to the *V. fischeri* protein (67% identical and 83% similar vs. 55–58% identical and 73% similar). Thus, future work assessing the requirements for SypE-mediated control of SypA can focus on the residues that are conserved in SypA and SypA_VP_ but not in RbdA. It remains possible that, while resistant to SypE, RbdA is still controlled via phosphorylation. While neither *V. parahaemolyticus* nor *V. vulnificus* contains a *sypE* homolog within their respective chromosomes, there are other predicted serine phosphatases and serine kinases that could potentially control the function of RbdA and SypA_VP_. Alternatively, perhaps in the pathogens there is no need to control the activity of SypA via phosphorylation, and the ability of SypE to control SypA_VP_ is an evolutionary artifact. We anticipate that the question of control by phosphorylation can be readily resolved by generating mutations in RbdA and SypA_VP_ that correspond to those that in SypA make its activity independent of SypE, such as S56A or R27A.

In summary, this work represents the most detailed structure/function analysis of a single STAS domain protein to date other than the paradigmatic anti-anti-sigma factor proteins of *Bacillus*. Our comparative and mutational analyses permitted us to identify key amino acids required for biofilm formation as well as for control by SypE. We anticipate that this work will provide an important foundation for the examination of SypA orthologs in other *Vibrio* species and a basis for understanding not only the function of this protein but also of other single STAS domain proteins that do not function as anti-anti-sigma factors.

## Conflict of Interest Statement

The authors declare that the research was conducted in the absence of any commercial or financial relationships that could be construed as a potential conflict of interest.
