# Corrigendum: Assessing the function of STAS domain protein SypA in *Vibrio fischeri* using a comparative analysis

**DOI:** 10.3389/fmicb.2017.00735

**Published:** 2017-04-26

**Authors:** Cecilia M. Thompson, Karen L. Visick

**Affiliations:** Department of Microbiology and Immunology, Loyola University ChicagoMaywood, IL, USA

**Keywords:** *Vibrio fischeri*, *Vibrio parahaemolyticus*, *Vibrio vulnificus*, biofilm formation, STAS domain proteins

Our work investigated the structure/function relationship of *V. fischeri* SypA using comparative analyses and mutagenesis approaches. We found that orthologs of SypA (RbdA, SypA_VP_) encoded by other *Vibrio* species (*V. vulnificus, V. parahaemolyticus*) were able to complement the biofilm defect of a *V. fischeri sypA* mutant. These results indicated that the function of these proteins is conserved. We also identified and disrupted a set of conserved residues in SypA; a number of these mutations diminished or abolished SypA function in promoting biofilm formation. Our conclusions with respect to these studies remain intact.

However, we also reported results with respect to control of SypA by SypE. In subsequent experiments, we discovered that two of our strains were incorrect. This affects some of our results and conclusions with respect to the data shown in Figures 5, 7. In Figure 5, we reported that strains expressing *rbdA* could largely overcome the inhibitory effect of SypE; however, we subsequently determined that this strain was incorrect as it did not express *sypE*. In the correct strain background, RbdA remains susceptible to control by SypE. Similarly, in Figure 7, we reported that a strain expressing a mutant form of SypA, SypA-R27A, was resistant to control by SypE, but that strain also proved to be incorrect as it did not express *sypE*. In the correct strain background SypA-R27A remains sensitive to SypE.

**Figure 5:**
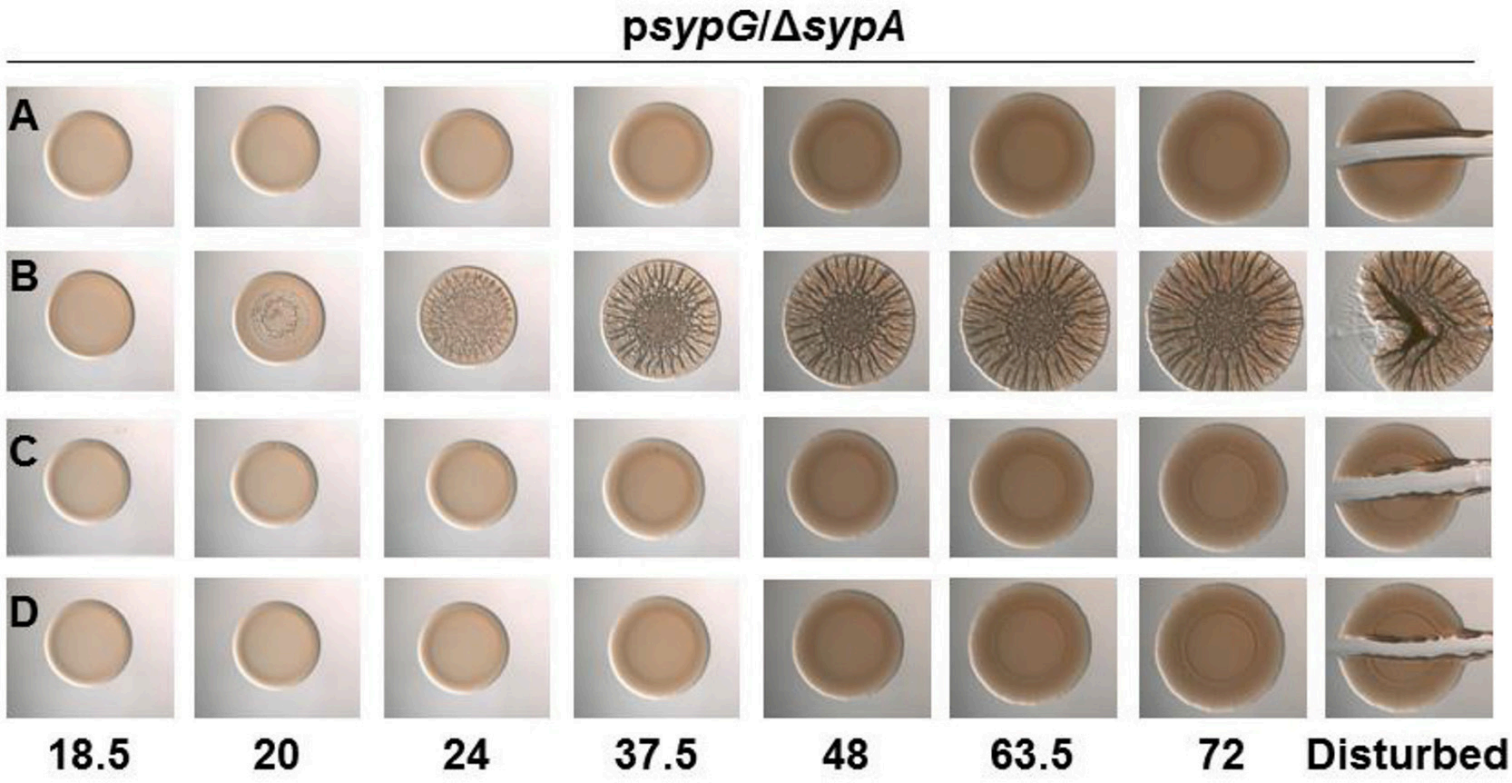
**SypA Proteins from Other Vibrios are Susceptible to Phosphorylation by *V. fischeri* SypE**. Development of colony morphology over time of *sypG* (pCLD56)-overexpressing derivatives of ΔsypA (sypE+) strains that contain **(A)** native *sypA* (KV5479), **(B)** sypA-S56A (expressing a mutant SypA that cannot be phosphorylated by SypE; KV5481), **(C)** *rbdA* (KV7309), or **(D)** *sypA*_vp_ (KV7313). Cultures were spotted onto LBS plates containing tet, and the morphologies of the resulting colonies were assessed at the indicated times. Representative images are shown. At 72 h, the colonies were disturbed with a toothpick to assess colony cohesiveness.

**Figure 7:**
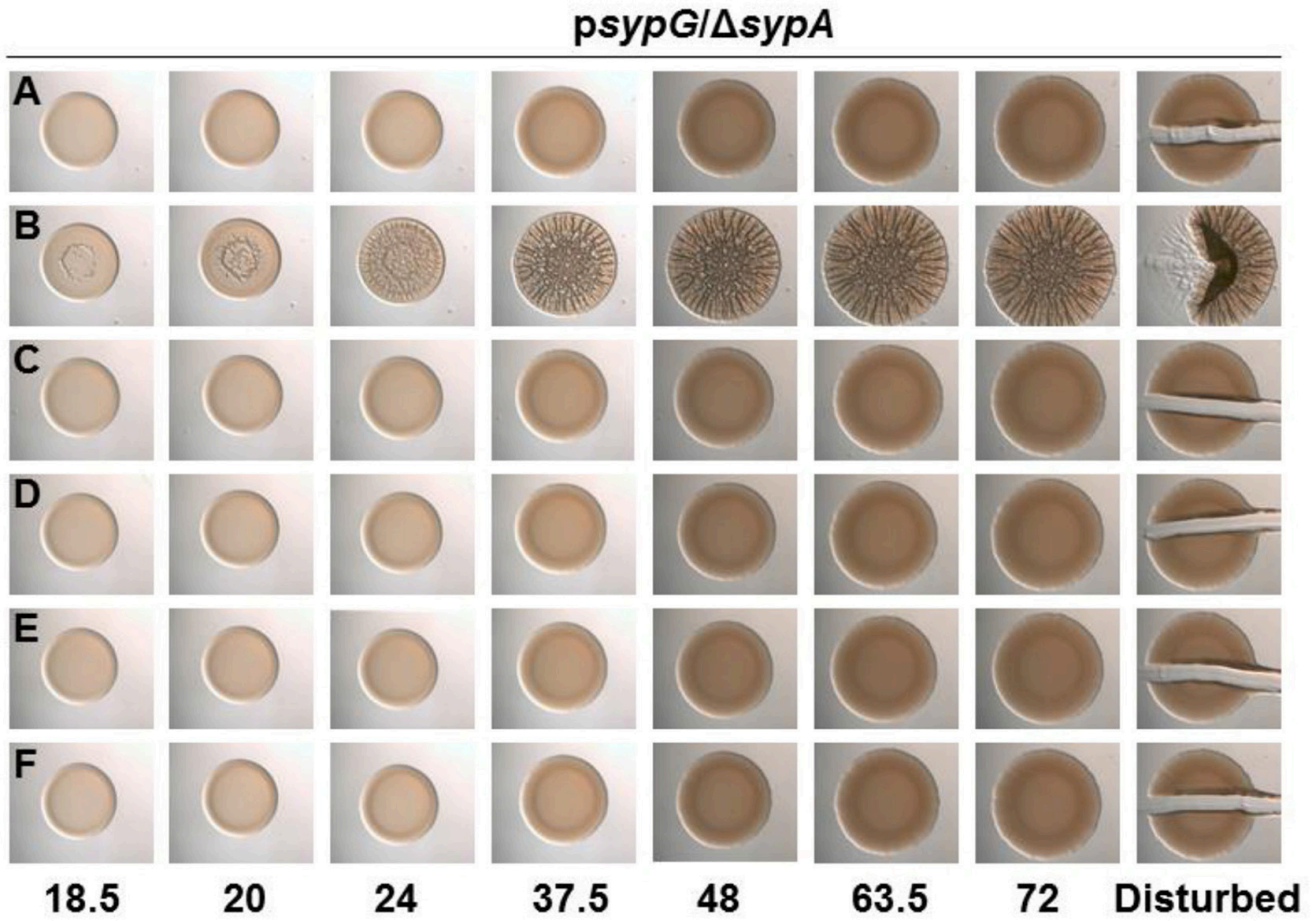
**All SypA mutants remain sensitive to control by SypE**. Development of colony morphology overtime of *sypG* (pCLD56)-overexpressing derivatives of Δ*sypA* (sypE+) strains that contain **(A)** native *sypA* (susceptible to SypE; KV6587), **(B)** *sypA*-S56A (expressing a mutant SypA that cannot be phosphorylated by SypE; KV6579), or the Class I mutants as follows: **(C)** *sypA*-G25A (KV7560), **(D)** *sypA*-E71A (KV7566), **(E)** *sypA*-R27A(KV7613), and **(F)** *sypA*-K72A(KV7616). Cultures were spotted onto LBS plates containing tet, and the morphologies of the resulting colonies were assessed at the indicated times. Representative images are shown.

We have repeated the pertinent experiments with strains that express SypE, and now provide an updated, revised version of the text (and corresponding figures) as follows:

The corrected section entitled “SypA Proteins from Other *Vibrios* are Susceptible to Phosphorylation by *V. fischeri* SypE” should read as follows:

The three proteins each contain a stretch of highly conserved residues with a serine residue; in SypA, this serine (S56) is phosphorylated by SypE (Figure 1) (Morris and Visick, 2013a,b). Although *sypE* is missing from the chromosome of *V. vulnificus* and *V. parahaemolyticus*, it is possible that RbdA and SypA_VP_ are also controlled via phosphorylation and may retain the ability to interact with and be inactivated by SypE from *V. fischeri*. To determine if RbdA and SypA_VP_ were susceptible to inactivation by SypE, we expressed the *sypA* orthologs in a *sypE*-containing *sypA* mutant (Δ*sypA sypE*^+^) and induced biofilm formation by overexpressing *sypG*. As expected, the negative control, a parent strain complemented with wild-type SypA fully susceptible to phosphorylation, failed to form wrinkled colonies, while the positive control, a parent strain complemented with SypA^S56A^, a mutant that cannot be inactivated via phosphorylation, formed robust wrinkled colonies in less than 24 h (Figures 5A,B). The colonies formed by the *rbdA*-containing strain failed to form wrinkled colonies, similar to the negative control. When we disrupted the colonies formed by *rbdA*-expressing *V. fischeri*, we found that the strain did not have cohesive properties, unlike the positive control (Figure 5C). Similarly, the *sypA*_VP_-expressing strain failed to form wrinkled or cohesive colonies even at later times (Figure 5D). These data indicate that SypA_VP_ and RbdA are both susceptible to regulatory control by *V. fischeri* SypE.

The corrected Figure 5 appears above.

The corrected section entitled “SypA Mutant is Resistant to Control by SypE” should have the following modified header and read as follows:

**All SypA mutants remain sensitive to control by SypE**. SypE binds to SypA and controls its activity via phosphorylation (Morris and Visick, 2013b). To date, Serine 56, the site of phosphorylation, is the only residue known to be critical for control by SypE. We hypothesized that other residues might facilitate the interaction between SypA and SypE, allowing for the phosphorylation of SypA, and that mutations in residues that facilitate this interaction would result in a SypA protein no longer recognized and/or phosphorylated by SypE. When introduced into a strain that expresses SypE, a mutant SypA that fails to interact with SypE will be “blind” to inhibition by SypE, resulting in biofilm formation under conditions in which it typically does not occur (e.g., *sypG* overexpression, Figure S1B). We thus expressed the four Class I *sypA* mutant alleles [those able to promote wrinkled colony formation similar to the positive control (Figure 6C)] in a strain that contained *sypE* and induced biofilm formation by overexpressing *sypG*. As expected, the negative and positive control strains failed to form and formed, respectively, wrinkled colonies (Figures 7A,B). Not unexpectedly, all of the four mutants failed to induce wrinkled colony formation, indicating that the mutant SypA protein remained susceptible to inhibition by SypE (Figures 7C–F). We hypothesize that we may be unable to disrupt SypE's ability to control SypA by making single mutations. It is possible that multiple mutations may need to be made in SypA to prevent contact and phosphorylation by SypE.

The corrected Figure 7 appears above.

## Conflict of interest statement

The authors declare that the research was conducted in the absence of any commercial or financial relationships that could be construed as a potential conflict of interest.
