# Supplementary material for: Assessing the function of STAS domain protein SypA in Vibrio fischeri using a comparative analysis
Source: Front Microbiol. 2015 Jul 28;6:760. doi: 10.3389/fmicb.2015.00760 (PMC4517449; doi:10.3389/fmicb.2015.00760)
Supplement: Supplementary file 1 [file Presentation_1.PDF]

Assessing the function of STAS domain protein SypA in *Vibrio fischeri* using a comparative analysis

Cecilia M. Thompson and Karen L. Visick*

Department of Microbiology and Immunology, Loyola University Chicago, Maywood, IL, USA

*** Correspondence:** Department of Microbiology and Immunology, Loyola University Chicago, 2160 S. First Ave. Bldg. 105, Rm 3933, Maywood, IL, 60153, USA.
kvisick@luc.edu

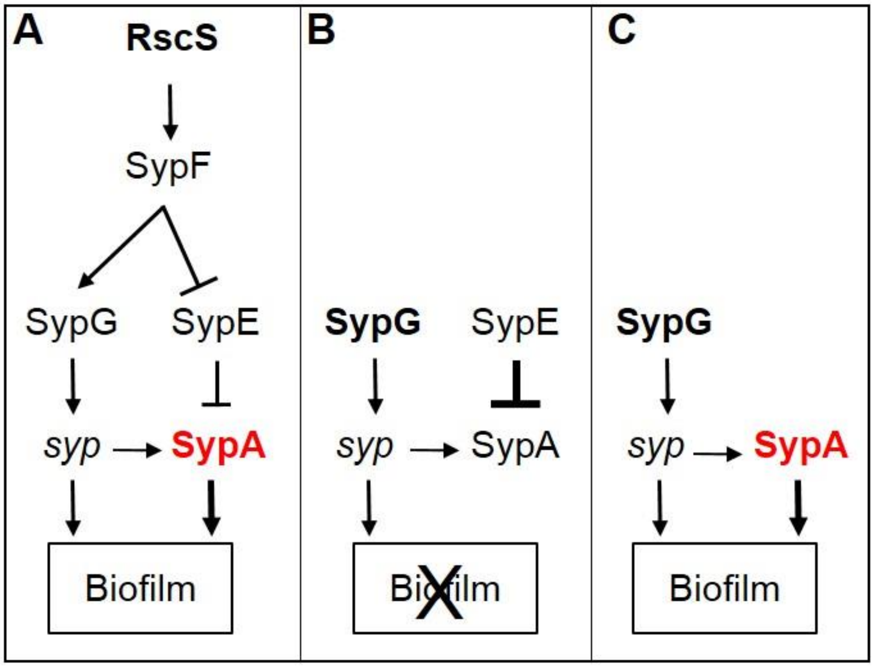

Figure S1. Model of biofilm formation by *Vibrio fischeri*. Biofilm formation depends upon the symbiosis polysaccharide (*syp*) locus and its regulators, including the sensor kinase RscS and the response regulator SypG, which activate *syp* transcription. It requires active, unphosphorylated SypA, which functions in an unknown way to promote biofilm formation. (A) When RscS is activated (or overexpressed), it activates, via SypF, the response regulator SypG to induce *syp* transcription. It also modulates the activity of the response regulator SypE: when phosphorylated, the activity of SypE is switched such that it functions as a serine phosphatase to dephosphorylate SypA, activating it to permit biofilm formation. (B) When SypG is overexpressed, it activates *syp* transcription. Under these conditions, however, unphosphorylated SypE functions as a serine kinase to phosphorylate SypA. Phosphorylated SypA is inactive and unable to induce biofilm formation. As a result, no biofilms form. (C) When SypG is overexpressed in the absence of SypE, SypA is not phosphorylated and is thus active to promote biofilm formation.

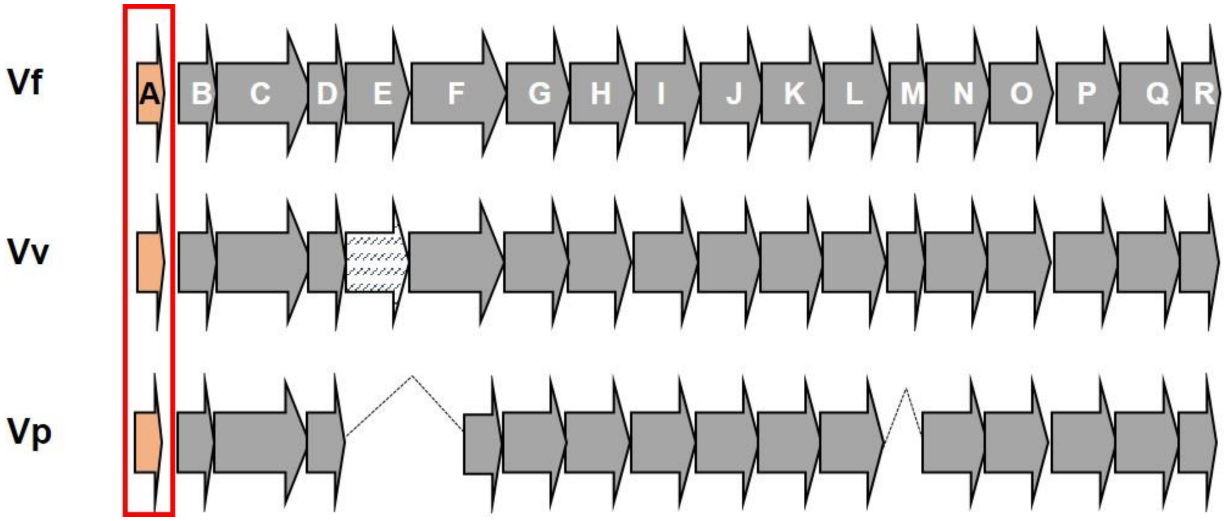

Figure S2. *syp* loci in *Vibrio* species. The 18-gene polysaccharide locus that has been shown in *V. fischeri* (Vf) to be involved in biofilm formation, shown at the top, is conserved in other *Vibrio* species. It can be found, among others, in the pathogens *V. parahaemolyticus* (Vp) and *V. vulnificus* (Vv) (Yip et al., 2005). The individual genes of the *V. vulnificus* and the *V. parahaemolyticus* loci have been only partially characterized (Kim et al., 2009;Guo and Rowe-Magnus, 2011;Ye et al., 2014). In all three organisms, the first gene in the locus encodes a STAS domain protein: SypA in *V. fischeri*, RbdA in *V. vulnificus*, and $SypA_{VP}$ in *V. parahaemolyticus*. Neither *V. parahaemolyticus* nor *V. vulnificus* contains a *sypE* gene: *V. vulnificus* carries an unrelated gene, *rbdE*, in the same position as *sypE*. *V. parahaemolyticus* lacks *sypE* and also encodes only the Hpt portion of SypF (Norsworthy and Visick, 2015).

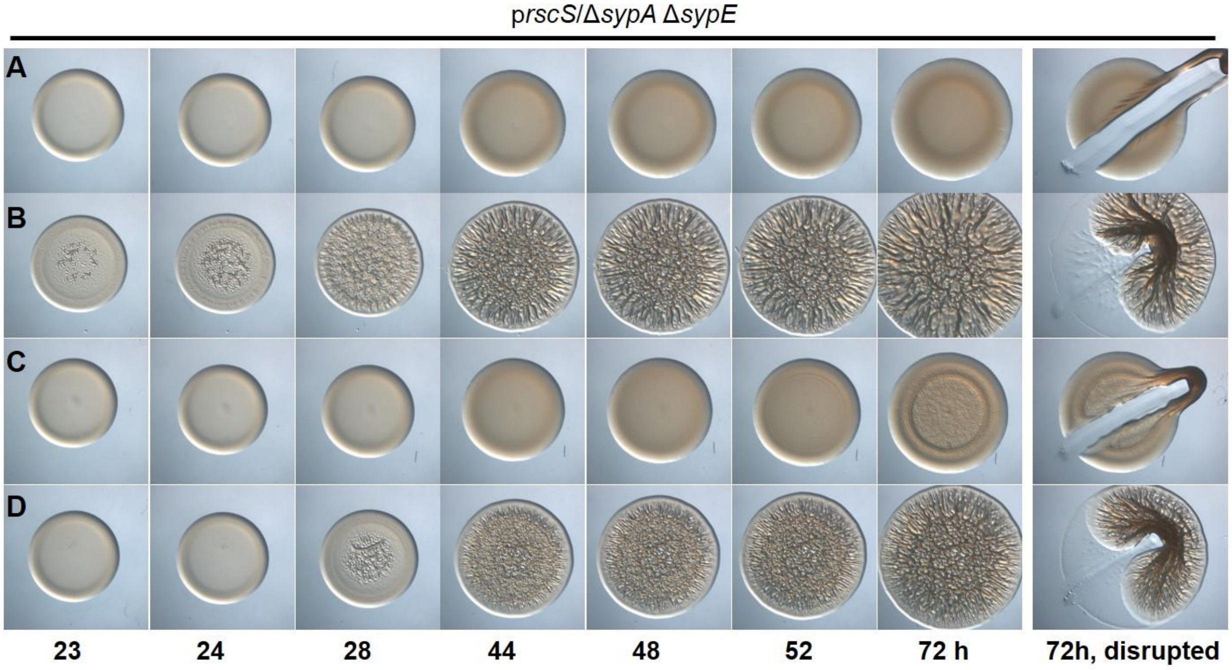

Figure S3. The absence of SypE impacts complementation by *rbdA* when RscS is overexpressed. Development of colony morphology over time of *rscS* (pARM7)-overexpressing derivatives of Δ*sypA* Δ*sypE* strains that contain (A) the empty cassette (negative control) (KV6392), (B) *sypA* (KV6393), (C) *rbdA* (KV7310), or (D) $sypA_{VP}$ (KV7314). Cultures were spotted onto LBS plates containing tet, and the morphologies of the resulting colonies were assessed at the indicated times. Representative images are shown. At 72 h, the colonies were disturbed with a toothpick to assess colony cohesiveness. Compare with Figure 2.

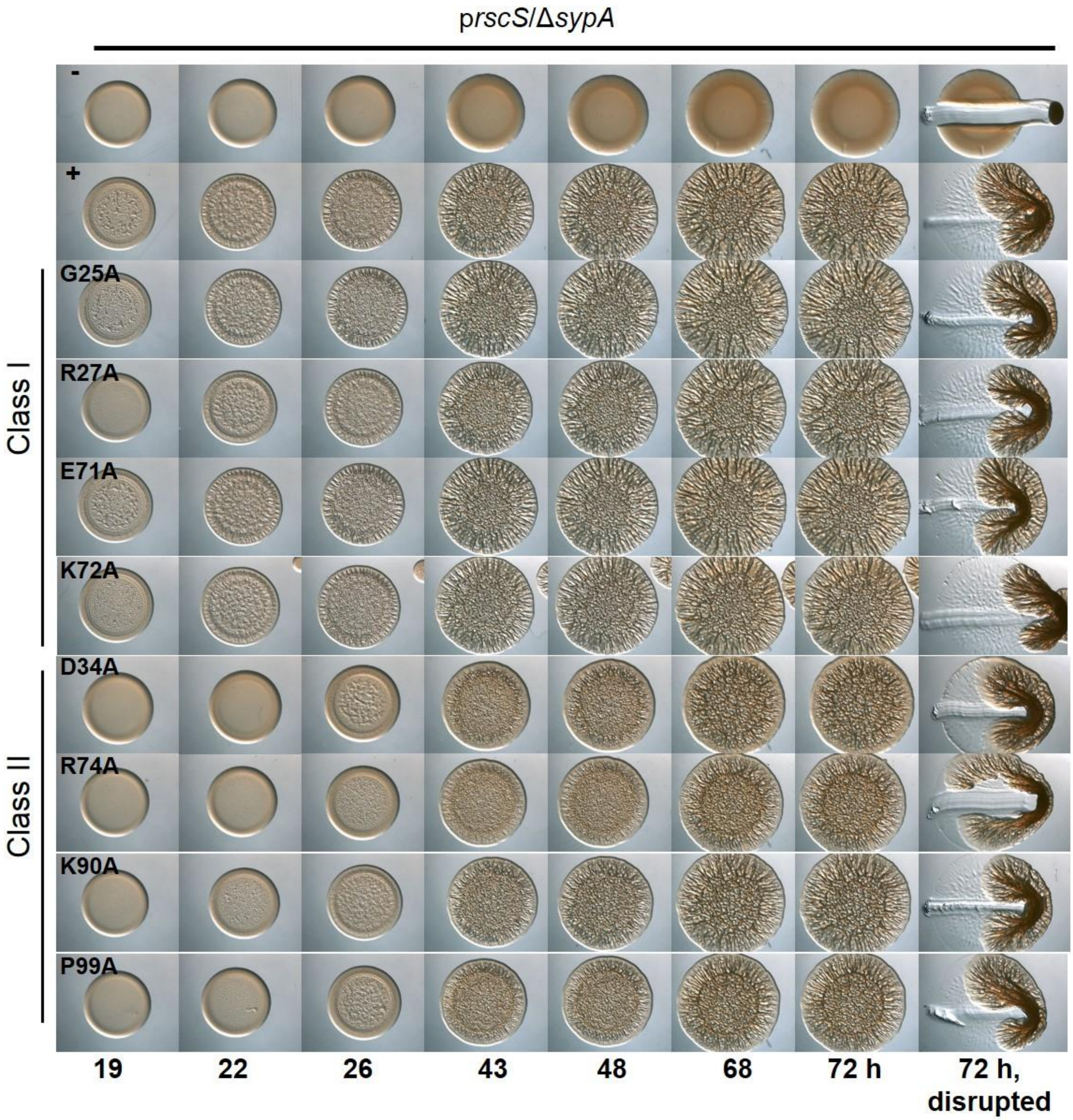
prscS/ΔsypA
-
+
G25A
R27A
E71A
K72A
D34A
R74A
K90A
P99A
Class I
Class II
19
22
26
43
48
68
72 h
72 h, disrupted

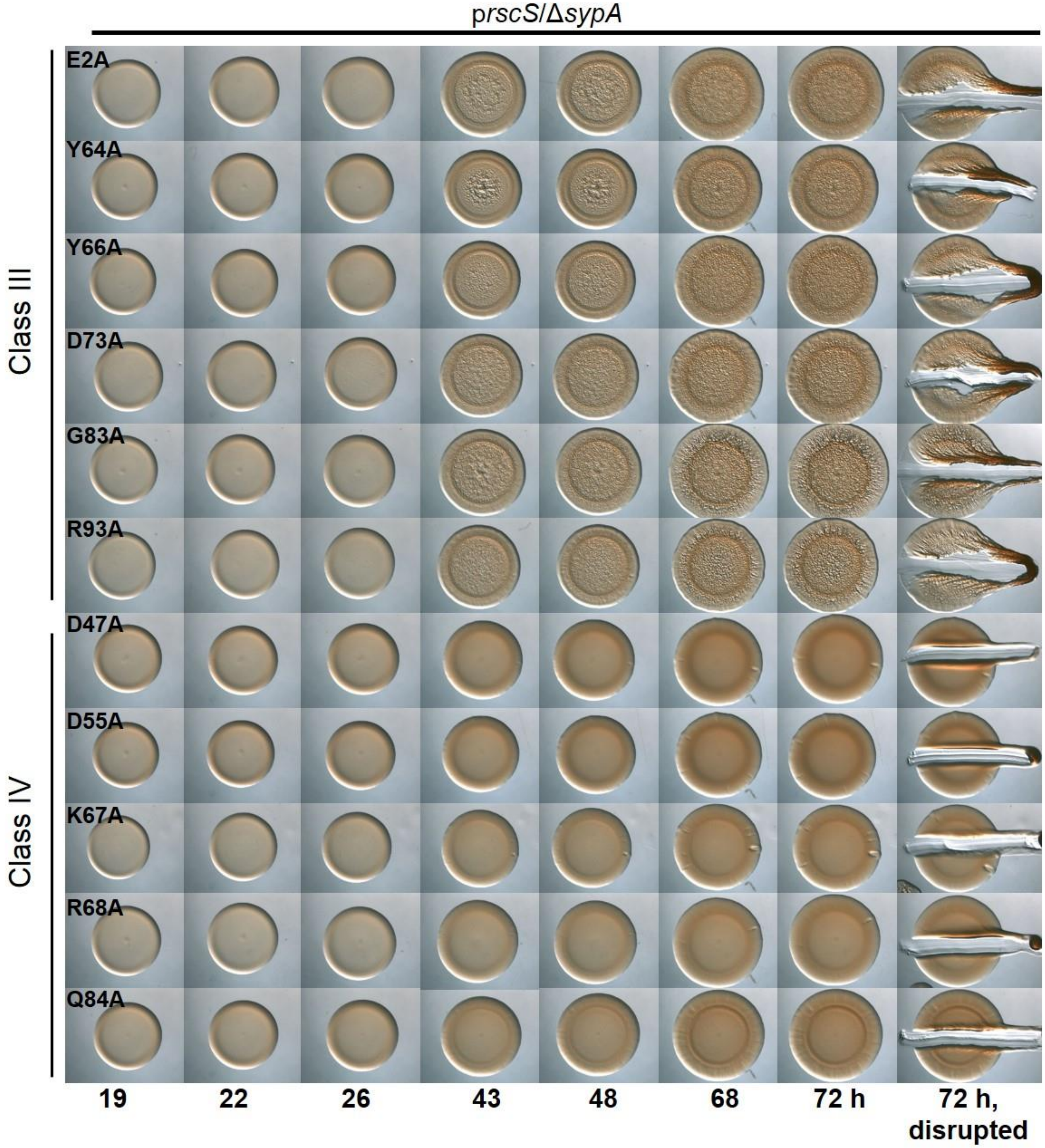
prscS/ΔsypA
Class III
Class IV
E2A
Y64A
Y66A
D73A
G83A
R93A
D47A
D55A
K67A
R68A
Q84A
19
22
26
43
48
68
72 h
72 h, disrupted

Figure S4. SypA point mutations exert a range of effects on biofilm formation. Development of colony morphology over time of *rscS* (pARM7)-overexpressing derivatives of Δ*sypA* strains that contain (-) the empty cassette (negative control) (KV5079), (+) *sypA* (KV6578), or *sypA* mutants with the indicated codon change. Mutant strains evaluated are as follows: G25A (KV7560); R27A (KV7613); E71A (KV7566); K72A (KV7616); D34A (KV7562); R74A (KV7568); K90A (KV7620); P99A (KV7572); E2A (KV7558); Y64A (KV7564); Y66A (KV7615); D73A (KV7612); G83A (KV7570); R93A (KV7010); D47A (KV7606); D55A (KV7607); K67A (KV6995); R68A (KV7005); Q84A (KV7000). Cultures were spotted onto LBS plates containing tet, and the morphologies of the resulting colonies were assessed at the indicated times. Representative images are shown. The images shown here for the negative (KV5079) and positive (KV6578) controls as well as those for *sypA* mutants *sypA*-R27A (KV7613), *sypA*-D34A (KV7562), *sypA*-R93A (KV7010), and *sypA*-K67A (KV6995) are the same as those shown in figure 6. Representative images are shown.

Supplemental table 1. Plasmids used in this study

Plasmids	Description[1]	Source or reference
pARM7	*rscS* overexpression plasmid; Tet^R	(Morris et al., 2011)
pARM47	Derivative of Tn*7* delivery plasmid pEVS107 that contains *sypE*, Kan^R, Erm^R	(Morris et al., 2011)
pARM163	Derivative of Tn*7* delivery plasmid pEVS107 that contains *sypA*-HA and its promoter	(Morris and Visick, 2013)
pCLD56	Derivative of low copy vector pKV282 that contains *sypG*	(Morris and Visick, 2013)
pCMT19	pARM47 containing *rbdA*	This study
pCMT21	pARM47 containing $sypA_{VP}$	This study
pCMT22	pARM47 containing $sypA_{VP}$-HA	This study
pCMT23	pARM47 containing $sypA^{E2A}$ HA	This study
pCMT24	pARM47 containing $sypA^{G25A}$ HA	This study
pCMT25	pARM47 containing $sypA^{D34A}$ HA	This study
pCMT26	pARM47 containing $sypA^{Y64A}$ HA	This study
pCMT27	pARM47 containing $sypA^{E71A}$ HA	This study
pCMT28	pARM47 containing $sypA^{R74A}$ HA	This study
pCMT29	pARM47 containing $sypA^{G83A}$ HA	This study
pCMT30	pARM47 containing $sypA^{P99A}$ HA	This study
pEVS104	tra^+ trb^+ *oriT*	(Stabb and Ruby, 2002)
pEVS107	Tn*7* delivery plasmid, Erm^R Kan^R	(McCann et al., 2003)
pKV282	Low copy vector, Tet^R	(Morris et al., 2011)
pKMG1	pARM47 containing $sypA^{D47A}$ HA	This study
pKMG2	pARM47 containing $sypA^{D55A}$ HA	This study
pKMG3	pARM47 containing $sypA^{D73A}$ HA	This study
pKMG4	pARM47 containing $sypA^{R27A}$ HA	This study
pKMG6	pARM47 containing $sypA^{Y66A}$ HA	This study
pKMG7	pARM47 containing $sypA^{K72A}$ HA	This study
pKMG11	pARM47 containing $sypA^{K90A}$ HA	This study
pSMM10	pARM47 containing $sypA^{K67A}$ HA	This study
pSMM11	pARM47 containing $sypA^{Q84A}$ HA	This study
pSMM12	pARM47 containing $sypA^{R68A}$ HA	This study
pSMM13	pARM47 containing $sypA^{R93A}$ HA	This study
pUX-BF13	Encodes Tn7 transposase	(Bao et al., 1991)

[1]All derivatives of pARM47 contain the indicated gene and lack the *sypE* gene; they also carry both the *lac* promoter and the *sypA* promoter.

Supplemental table 2. Oligonucleotides used in this study

Primer	Sequence (5’ -3’)
sypA Gibson F	GATTACGCCAAGCTTGCATGC
sypA Gibson R	CAGTCTAGTTCTAGAGGGCCC
sypA K67A F	TTATCTATATGCACGACTTATAGAGAAAGATCGTA
sypA K67A R	CTATAAGTCGTGCATATAGATAAACAATAGCGCCAA
sypA Q84A F	TGCACATGGCGCGCCACTAGAGTTACTAAAACTTC
sypA Q84A R	ACTCTAGTGGCGCGCCATGTGCATTTTTAATCTGC
sypA R68A F	TCTATATAAAGCACTTATAGAGAAAGATCGTACTAT
sypA R68A R	TCTCTATAAGTGCTTTATATAGATAAACAATAGCGCC
sypA R93A F	AAAACTTCTAGCCATTGAAAACGCAATTCCTGTTAA
sypA R93A R	CGTTTTCAATGGCTAGAAGTTTTAGTAACTCTAGTG
VV *sypA* F	GATTACGCCAAGCTTGCATGCAACAGGAGAACGTCAC
VV *sypA* R	CAGTCTAGTTCTAGAGGGCCCTTTATCATCATCATCTTTATAATCCTAAAACTGCTTGGAGTT
VP *sypA* F	GATTACGCCAAGCTTGCATGCTAAATGGAGATAGGGTC
VP *sypA* R	CAGTCTAGTTCTAGAGGGCCCTTTATCATCATCATCTTTATAATCTTAGTGTCCTTTTGAATTG
VP *sypA*-HA R	CAGTCTAGTTCTAGAGGGCCCTTATGCATAATCTGGAACATCATATGGATAGTGTCCTTTTGAATTG
VV *sypA* Promoter R	GTGACGTTCTCCTGTTGCATGCGCTCCTAGGGAATAATCC
VP *sypA* Promoter R	GACCCTATCTCCATTTAGCATGCGCTCCTAGGGAATAATCC
sypA E2A F	GCTTATTATGGCACTACATCAATTCGAATCAAATGA
sypA E2A R	ATTGATGTAGTGCCATAATAAGCTCCTAGGGAATA
sypA G25A F	GGACGCCATCGCATGTAGAGATATTCAACCATCCA
sypA G25A R	TATCTCTACATGCGATGGCGTCCATATCACCTT
sypA D34A F	ACCATCCATCGCAAGCGTGATTGAACAAGAACATC
sypA D34A R	CAATCACGCTTGCGATGGATGGTTGAATATCTCTA
sypA Y64A F	CGCTATTGTTGCACTATATAAACGACTTATAGAGAAA
sypA Y64A R	GTTTATATAGTGCAACAATAGCGCCAATACCTGA
sypA E71A F	ACGACTTATAGCAAAAGATCGTACTATGCAGATTAA
sypA E71A R	TACGATCTTTTGCTATAAGTCGTTTATATAGATAAACA
sypA R74A F	AGAGAAAGATGCAACTATGCAGATTAAAAATGCACA
sypA R74A R	TCTGCATAGTTGCATCTTTCTCTATAAGTCGTTTATA
sypA G83A F	AAATGCACATGCACAGCCACTAGAGTTACTAAAAC
sypA G83A R	CTAGTGGCTGTGCATGTGCATTTTTAATCTGCATAG
sypA P99A F	AAACGCAATTGCAGTTAATAAAACAACGCATTATCC
sypA P99A R	TTTTATTAACTGCAATTGCGTTTTCAATACGTAGAA
sypA R27A F	CATCGGTTGTGCAGATATTCAACCATCCATCGATAG
sypA R27A R	GTTGAATATCTGCACAACCGATGGCGTCCATATC
sypA D47A F	AGTTCAAATCGCATTATCACACGTAGCCTTTTTAG
sypA D47A R	CGTGTGATAATGCGATTTGAACTTGATGATGTTC
sypA D55A F	AGCCTTTTTAGCATCATCAGGTATTGGCGCTATTG
sypA D55A R	TACCTGATGATGCTAAAAAGGCTACGTGTGATAA
sypA Y66A F	TGTTTATCTAGCAAAACGACTTATAGAGAAAGATCG
sypA Y66A R	TAAGTCGTTTTGCTAGATAAACAATAGCGCCAATACC
sypA K72A F	ACTTATAGAGGCAGATCGTACTATGCAGATTAAA
sypA K72A R	TAGTACGATCTGCCTCTATAAGTCGTTTATATAG
sypA D73A F	TATAGAGAAAGCACGTACTATGCAGATTAAAAATGC
sypA D73A R	GCATAGTACGTGCTTTCTCTATAAGTCGTTTATATAG
sypA K90A F	AGAGTTACTAGCACTTCTACGTATTGAAAACGCA
sypA K90A R	TACGTAGAAGTGCTAGTAACTCTAGTGGCTGGCC

Supplemental references

Bao, Y., Lies, D.P., Fu, H., and Roberts, G.P. (1991). An improved Tn*7*-based system for the single-copy insertion of cloned genes into chromosomes of Gram-negative bacteria. *Gene* 109**,** 167-168.

Guo, Y., and Rowe-Magnus, D.A. (2011). Overlapping and unique contributions of two conserved polysaccharide loci in governing distinct survival phenotypes in *Vibrio vulnificus*. *Environ Microbiol* 13**,** 2888-2990. doi: 10.1111/j.1462-2920.2011.02564.x.

Kim, H.S., Park, S.J., and Lee, K.H. (2009). Role of NtrC-regulated exopolysaccharides in the biofilm formation and pathogenic interaction of Vibrio vulnificus. *Mol Microbiol* 74**,** 436-453. doi: 10.1111/j.1365-2958.2009.06875.x.

Mccann, J., Stabb, E.V., Millikan, D.S., and Ruby, E.G. (2003). Population dynamics of *Vibrio fischeri* during infection of *Euprymna scolopes*. *Appl Environ Microbiol* 69**,** 5928-5934.

Morris, A.R., Darnell, C.L., and Visick, K.L. (2011). Inactivation of a novel response regulator is necessary for biofilm formation and host colonization by *Vibrio fischeri*. *Mol Microbiol* 82**,** 114-130. doi: 10.1111/j.1365-2958.2011.07800.x.

Morris, A.R., and Visick, K.L. (2013). Inhibition of SypG-induced biofilms and host colonization by the negative regulator SypE in *Vibrio fischeri*. *PLoS One* 8**,** e60076. doi: 10.1371/journal.pone.0060076.

Norsworthy, A.N., and Visick, K.L. (2015). Signaling between two interacting sensor kinases promotes biofilms and colonization by a bacterial symbiont. *Mol Microbiol*. doi: 10.1111/mmi.12932.

Stabb, E.V., and Ruby, E.G. (2002). RP4-based plasmids for conjugation between *Escherichia coli* and members of the Vibrionaceae. *Methods Enzymol* 358**,** 413-426.

Ye, L., Zheng, X., and Zheng, H. (2014). Effect of *sypQ* gene on poly-N-acetylglucosamine biosynthesis in *Vibrio parahaemolyticus* and its role in infection process. *Glycobiology* 24**,** 351-358. doi: 10.1093/glycob/cwu001.

Yip, E.S., Grublesky, B.T., Hussa, E.A., and Visick, K.L. (2005). A novel, conserved cluster of genes promotes symbiotic colonization and σ^{54}-dependent biofilm formation by *Vibrio fischeri*. *Mol Microbiol* 57**,** 1485-1498. doi: 10.1111/j.1365-2958.2005.04784.x.
